# The ubiquitin-conjugating enzyme, Ubc1, indirectly regulates SNF1 kinase activity via Forkhead-dependent transcription

**DOI:** 10.15698/mic2016.11.538

**Published:** 2016-11-04

**Authors:** Rubin Jiao, Liubov Lobanova, Amanda Waldner, Anthony Fu, Linda Xiao, Troy A. Harkness, Terra G. Arnason

**Affiliations:** 1 Department of Anatomy and Cell Biology, University of Saskatchewan, Saskatoon, SK, Canada S7N 5E5.; 2 Department of Medicine, University of Saskatchewan, Saskatoon, SK, Canada S7N 5E5.

**Keywords:** SNF1 kinase, Ubc1, Forkheads, APC, protein stability

## Abstract

The SNF1 kinase in *Saccharomyces cerevisiae* is an excellent model to study the regulation and function of the AMP-dependent protein kinase (AMPK) family of serine-threonine protein kinases. Yeast discoveries regarding the regulation of this non-hormonal sensor of metabolic/environmental stress are conserved in higher eukaryotes, including poly-ubiquitination of the α-subunit of yeast (Snf1) and human (AMPKα) that ultimately effects subunit stability and enzyme activity. The ubiquitin-cascade enzymes responsible for targeting Snf1 remain unknown, leading us to screen for those that impact SNF1 kinase function. We identified the E2, Ubc1, as a regulator of SNF1 kinase function. The decreased Snf1 abundance found upon deletion of Ubc1 is not due to increased degradation, but instead is partly due to impaired SNF1 gene expression, arising from diminished abundance of the Forkhead 1/2 proteins, previously shown to contribute to *SNF1* transcription. Ultimately, we report that the Fkh1/2 cognate transcription factor, Hcm1, fails to enter the nucleus in the absence of Ubc1. This implies that Ubc1 acts indirectly through transcriptional effects to modulate SNF1 kinase activity.

## INTRODUCTION

The SNF1 kinase class of serine/threonine kinases, which includes the AMP-dependent protein kinase (AMPK) in other systems, are of widespread interest because of their important roles in glucose homeostasis, stress resistance, and aging 1,2,3. These enzymes are inactive under optimal conditions, yet are rapidly activated in response to a wide variety of nutritional and stress cues. The active kinases, in turn, exert their activity to alter cellular pathways at the protein and transcriptional level to maintain homeostasis or to direct adaptive mechanisms for stress resistance. Simply, low glucose growth conditions will activate SNF1 kinase in yeast, whereas muscle contraction or fasting will do the same in animals 3,4,5. The dominant, essential, and finely responsive regulatory step is the phosphorylation of Snf1 by upstream kinases, balanced by its controlled dephosphorylation 4,6. This governing event is conserved between yeast and all higher eukaryotes. In yeast, SNF1 kinase directly phosphorylates a variety of downstream targets, including the nuclear target Mig1 7 and the cytosolic target Rod1 8. Mig1 functions as a transcriptional inhibitor that prevents *SUC2* expression, whereas Rod1 is involved in glucose transporter endocytosis 8. Several other described events act on SNF1 kinase including allosteric tightening of the Snf1 (α) and Snf4 (γ) subunits within the heterotrimeric complex (by convention, note lower case for the yeast protein α subunit [Snf1], upper case for the enzyme complex [SNF1 kinase]) 9, and the nuclear accumulation of the Snf1-Gal83 (β subunit)-Snf4 enzyme within the nucleus, necessary for transcriptional changes 10. In addition, our recent work reported that the yeast orthologs of mammalian Forkhead Box (Fox) transcription factors, Forkhead (Fkh) 1 and 2, regulate *SNF1* gene transcription, impacting the protein abundance of the Snf1 subunit 11. The Snf1 subunit contains autoinhibitory and ubiquitin-associated (UBA) domains that act as restraining functions on activity 11,12. More recently, additional negative influences have been shown to occur through Snf1 subunit posttranslational modifications, specifically ubiquitination and SUMOylation, which effectively decreased Snf1 protein abundance through degradation, with resulting reductions in SNF1 kinase activity 13,14,15. Ubiquitin (Ub) becomes covalently attached to target proteins through the sequential action of Ub activating (E1), conjugating (E2), and ligase (E3) activities: in yeast there is a single E1, a finite well-described group of eleven E2s, and an ever-expanding recognition of E3 ligase activities.

Our goal was to identify discrete E2s that are involved in SNF1 kinase activity in response to glucose levels and anticipated revealing those which are involved in Snf1-Ub attachment. Here, we report that the cell cycle and stress-related E2, Ubc1, indirectly affects SNF1 kinase activity not through stability, but through upstream events effecting the yeast Fox orthologs Fkh1/2 that provide transcriptional control of the Snf1 subunit. Ubc1 is known to act, along with Ubc4, with the anaphase promoting complex (APC) to target and polyubiquitinate APC substrates for cell cycle related degradation to enable exit from metaphase, and entry to G1 15. Intriguingly, the *ubc1*∆ mitotic arrest point is consistent with a disruption of APC-dependent exit from mitosis and failure to enter G1 16. Nonetheless, our data suggest that Ubc1 acts on Hcm1 in an APC-independent manner. Our observations suggest a model where Hcm1 is modified in a Ubc1- dependent manner to facilitate Hcm1 nuclear shuttling and activation of the Fkh/Snf1 stress response pathway.

## RESULTS

### Deletion of the E2 enzyme, Ubc1, impairs SNF1 kinase-dependent invertase activity.

When glucose is limiting, yeast adapts to using alternative carbon sources. A key function of the SNF1 kinase is to adapt metabolic pathways to non-glucose carbohydrate sources, and the mechanism is particularly well documented for sucrose. Sucrose utilization requires the expression of invertase, an enzyme that cleaves the disaccharide sucrose molecule into glucose and fructose, which is encoded by the *SUC2* gene 7. When glucose is abundant, *SUC2* expression is repressed by the binding of the transcriptional repressor, Mig1 to the *SUC2* promotor. Under limiting glucose conditions, activated SNF1 kinase enters the nucleus, phosphorylates Mig1 protein via its inherent kinase activity and releases Mig1 from the *SUC2* promoter. The subsequent expression of *SUC2* can be quantitatively determined by colorimetric “Invertase assay” 18 or directly through RT-PCR, as an indirect measure of SNF1 kinase activity.

**Figure 1:**
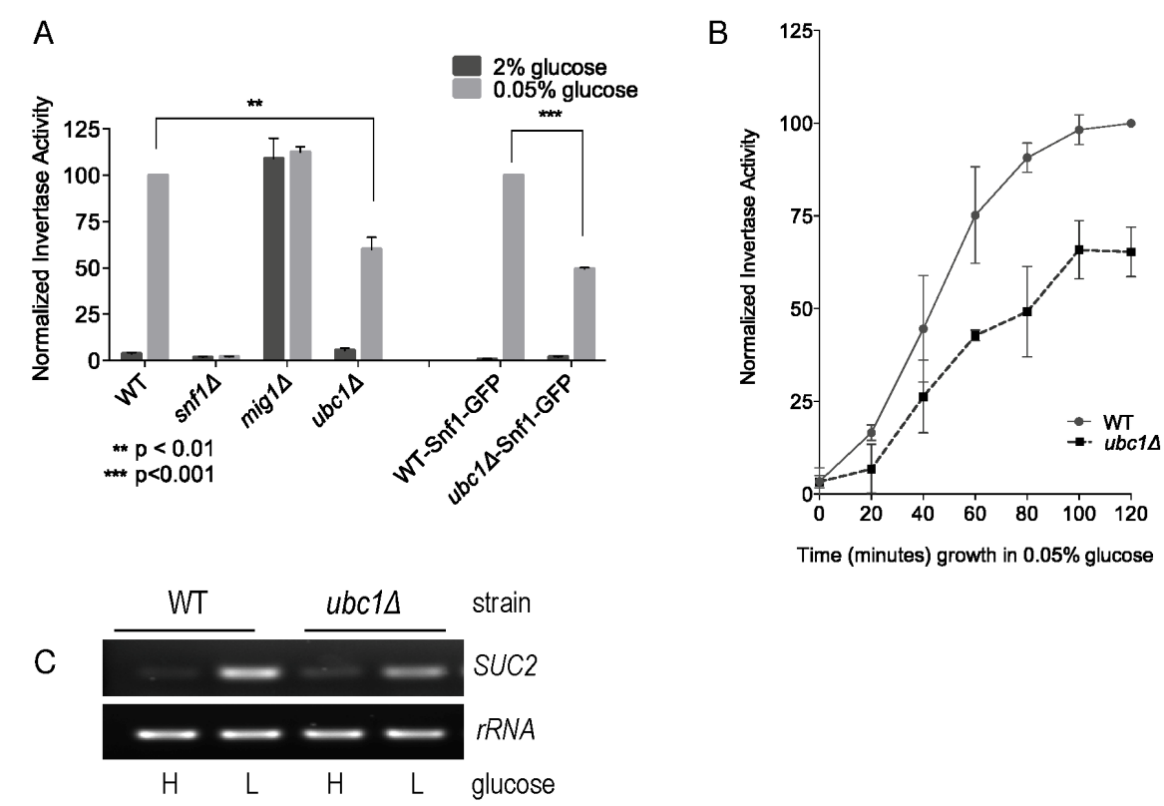
FIGURE 1: Yeast strains deleted for the ubiquitin conjugating enzyme, Ubc1, are impaired for SNF1 kinase-dependent invertase activity expressed from the *SUC2* gene. Comparison of invertase activity under repressive (2% glucose) and activating (0.05% glucose) conditions between isogenic yeast strains normalized to WT in 0.05% glucose (value of 100), showing the mean and SD. WT-Snf1-GFP and *ubc1*Δ-Snf1-GFP have a genomic green-fluorescent protein (GFP) sequence integrated in-frame with the endogenous Snf1 sequence. **(A)** Invertase activity after 2 hours of activating growth conditions. Statistical significance based on 3 biological repeats using t-test (Prism 6.0). **(B)** Chronological invertase activity of WT and *ubc1*Δ strains, sampled intermittently over 2 hours following shift to low glucose media. Average of four biological repeats with SEM are indicated for each time point. **(C)** Agarose gel of RT-PCR products (26 cycles) using primers against *SUC2* and *rRNA* loci from RNA isolated from WT and *ubc1*Δ strains grown in 2% (H: high) and 0.05% (L: low, 2 hours) glucose.

Fig. 1A demonstrates the expected rise in invertase activity after 2 hours of growth in low (0.05%) glucose in a wild type (WT) yeast strain, and the complete dependence of this on the Snf1 α catalytic subunit (*snf1*Δ). In contrast, disruption of the *SUC2* transcriptional repressor Mig1 (*mig1*Δ) 7 demonstrates high activity regardless of glucose levels, as expected for loss of regulated repression. Disruption of the *UBC1* gene (*ubc1*Δ) resulted in a significant decrease in invertase activity as compared to WT. This defect was also observed in WT and *ubc1*Δ strains carrying the endogenous green-fluorescent (GFP) protein Snf1-GFP fusion into either strain (WT-Snf1-GFP or *ubc1*Δ-Snf1-GFP). To expand on this, we also tested invertase activity at short intervals leading up to a 2 hour time point (Fig. 1B) and report the presence of early (1 hour) and sustained impairment of invertase activity. The corresponding changes to *SUC2* expression in the WT and *ubc1*Δ strains under activating and repressive conditions paralleled that of the invertase RNA (Fig. 1C).

### Loss of Ubc1 function does not impair SNF1 kinase nuclear accumulation, allosteric associations or substrate targeting.

**Figure 2:**
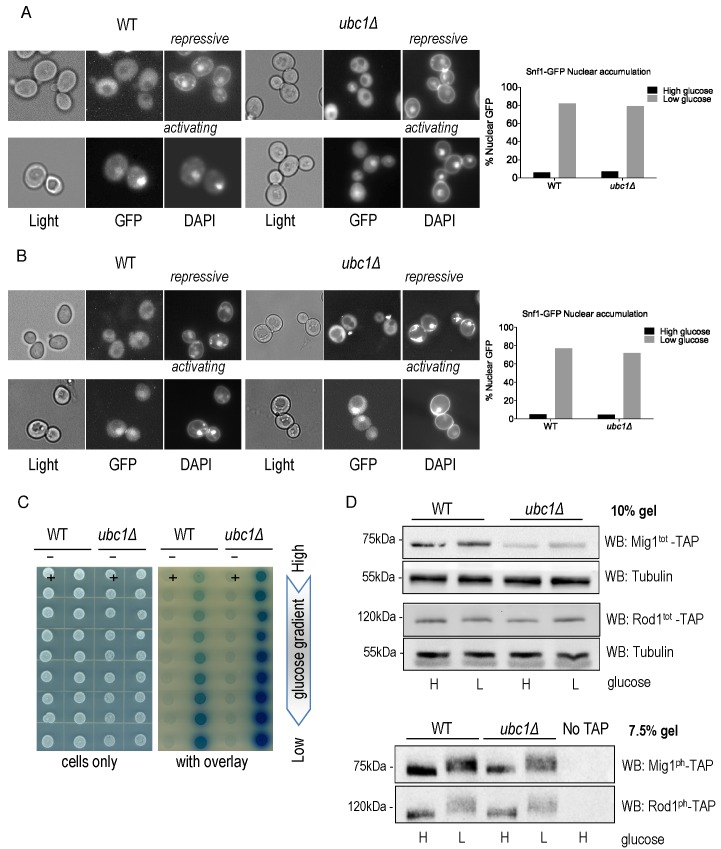
FIGURE 2: Loss of Ubc1 function does not impair SNF1 kinase nuclear accumulation, allosteric associations or substrate targeting. **(A)** Fluorescent microscopy of GFP-tagged yeast Snf1 constitutively expressed from a high copy 2μ plasmid or **(B)** expressed from the endogenous Snf1 promoter in isogenic WT and *ubc1*Δ strains grown under repressive (2% glucose) or shifted to activating (5% glycerol for 30 minutes) conditions. The percent of cells with Snf1-GFP nuclear accumulation was quantitated from four biological repeats. 125 consecutive cells were scored for co-localization of GFP and DAPI signal in high and low glucose in the isogenic WT and *ubc1*Δ strains. **(C)** 2-hybrid associations between empty vectors (-) and Snf1-Snf4 pairs (+) in WT and *ubc1*Δ strains are shown. Equal cell numbers were spotted down a glucose gradient (0.05% to 2% glucose) before and after β-galactosidase color development from overlay. **(D)** Isogenic WT and *ubc1*Δ strains harboring endogenous TAP-epitope tags to Mig1 and Rod1 were divided into high (H: 2%) and low (L: 0.05%) glucose media for 30 minutes prior to cell lysis. Duplicate sample were run in parallel on 10% acrylamide gels (for total TAP-protein; Mig1^tot^ and Rod1^tot^, upper panels) and 7.5% (to enhance the phospho-shift; Mig1^ph^ and Rod1^ph^, lower panels). WB: Western Blot primary antibody/target. Light: non fluorescent 100 x objective. GFP: green fluorescent protein epitope tag. DAPI: (4',6-Diamidino-2-Phenylindole, Dihydrochloride) a fluorescent DNA interchelator.

Invertase assay defects (or decreases in maximal *SUC2* expression) can arise from disruption of any of multiple steps in SNF1 kinase activation, including protein abundance, activating phosphorylation, allosteric associations, nuclear import, or phosphorylation of Mig1. These stages can be isolated and independently assessed to pinpoint where Ubc1 affects SNF1 kinase activity. First, we asked if Ubc1 was required for movement of the Snf1-Gal83-Snf4 kinase complex into the nucleus under activating conditions, as a failure to efficiently accumulate in the nucleus would explain the impairment in transcriptional release of *SUC2* expression. We initially expressed Snf1-GFP constitutively from a 2μ yeast plasmid transformed into WT and *ubc1*Δ strains and used live fluorescent microscopy of these isogenic strains to localize Snf1-GFP to the nucleus (identified by DAPI staining) (Fig. 2A). The plasmid-expressed Snf1 subunit rapidly relocates to the nucleus after stimulating conditions in a manner indistinguishable from WT 10. Furthermore, endogenous expression of a genomic version of GFP-tagged Snf1 subunit did not alter the efficiency of nuclear accumulation in WT or *ubc1*Δ isogenic strains (Fig. 2B). Multiple biological repeats of these experiments allowed us to score the relative efficiency of nuclear import of Snf1-GFP in these WT and *ubc1*Δ strains, demonstrating that there is no impairment of nuclear accumulation in the absence of the functional Ubc1 protein.

Next, we assessed if the *UBC1* disruption interfered with the allosteric associations between the α and γ subunits of the kinase upon activation. 2-hybrid analysis was used to compare Snf1-Snf4 interactions throughout a glucose gradient; β-galactosidase production results in a visible blue color and correlates with the strength of the associations between these two proteins 11. In WT yeast, we observed increased Snf1-Snf4 associations within the 2-hybrid assay as the glucose concentration drops, which is also seen in the isogenic *ubc1*Δ strain, at even greater levels than WT (Fig. 2C). Clearly, the invertase defect linked with *UBC1* disruption is not related to allosteric hindrance.

We next asked if the *UBC1* deletion affected the ability of SNF1 kinase to phosphorylate known protein targets. De-repression of *SUC2* requires SNF1 kinase-dependent phosphorylation of the nuclear transcriptional repressor protein, Mig1. Non-nuclear targets for SNF1 kinase phosphorylation include the Rod1 protein that resides at the plasma membrane 8. Glucose responsive phosphorylation of both nuclear Mig1 and cytosolic Rod1 can be directly assessed by Western analysis by their visible phospho-shift to higher molecular weights 7,8. We find a noticeable upwards phospho-shift of each target under low glucose in both WT and *ubc1*Δ strains (Fig. 2D). Although there is an apparent decrease in Mig1 protein abundance in the *ubc1*Δ strain, the phospho-shift is not impaired, nor is there a defect in the expected Mig1 nuclear export under activating conditions (Fig. S1). Therefore, Ubc1 deletion does not impair the enzymatic activity of SNF1 kinase.

### Snf1 protein abundance, but neither stability nor phosphorylation, is decreased by Ubc1 disruption.

**Figure 3:**
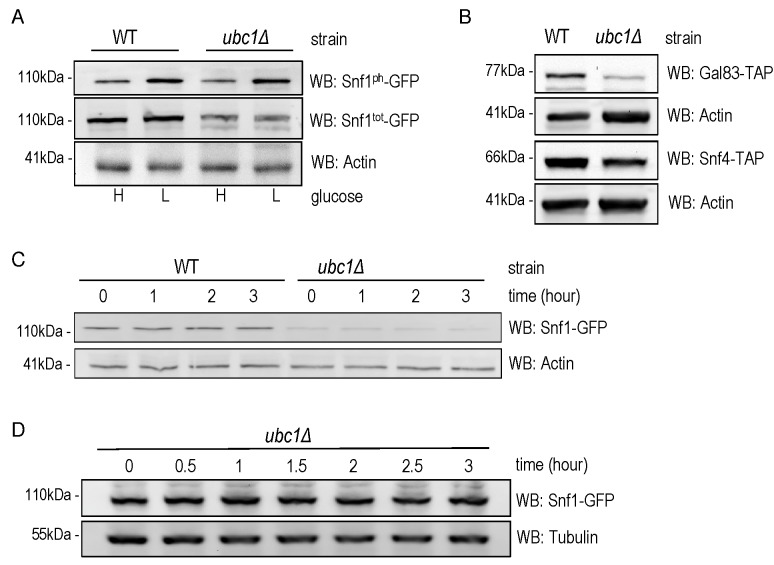
FIGURE 3: Snf1 protein abundance, but neither stability nor phosphorylation, is decreased by Ubc1 disruption. **(A)** Early logarithmic phase WT and *ubc1*Δ strains harboring genomic Snf1-GFP were grown in 2% (H) and 0.05% (L) glucose for 1 hour prior to cell lysis, and an equal amount of total protein was loaded in duplicates for Western analysis of total Snf1 (Snf1^tot^-GFP) and phosphorylated Snf1 (Snf1^ph^-GFP). **(B)** WT and *ubc1*Δ strains harboring genomic Gal83-TAP or Snf4-TAP were treated as in (A) and Western analysis for TAP abundance is shown. **(C)** Assessment of Snf1 protein stability over 3 hours in WT and *ubc1*Δ strains in the presence of cycloheximide, added at time (0), in 2% glucose. Equal cell numbers were removed at the indicated time points with 40 μg protein loaded. **(D)** Biological repeat of Snf1-GFP stability (as in B) is shown with 80 μg protein loaded per lane, and additional timepoints.

An obvious question to ask was whether the role of Ubc1 in SNF1 kinase regulation was to target Snf1 for ubiquitination, and ultimately degradation. It is known that the catalytic Snf1 α-subunit can be polyubiquitinated and thereby effect its abundance 14. We compared the steady-state abundance of endogenous Snf1-GFP protein in logarithmically growing WT and *ubc1*Δ strains (Fig. 3A) and observed a clear decrease in Snf1 abundance, irrespective of the activation state of the enzyme, limited to the *ubc1*Δ strain. This is not consistent with Ubc1-dependent ubiquitination and subsequent degradation of Snf1, as this would instead manifest as an increase in Snf1 protein in the *UBC1* deletion. Activating Snf1 phosphorylation was maintained at near-WT levels despite the decrease in total Snf1 protein (Fig. 3A). In addition to Snf1, protein levels of both the endogenous Snf4 γ- and Gal83 β-subunits were down in the *ubc1*Δ strain, compared to WT (Fig. 3B). To directly assess if there was enhanced degradation of Snf1 in the absence of Ubc1 function, we performed cycloheximide protein degradation assays of Snf1 over a 3-hour period (Fig. 3C) in WT and *ubc1*Δ strains. Snf1 was stable over the three-hour period in the WT strain and also appeared stable in *ubc1*Δ. To confirm the stability of Snf1 in the *ubc1*Δ strain, a biological repeat was performed with a greater protein load (80 μg/lane versus 40 μg/lane) (Fig. 3D). These results suggest that Ubc1 does not play a role in Snf1 protein stability.

### *SNF1* expression and Fkh protein abundance are decreased upon Ubc1 deletion.

**Figure 4:**
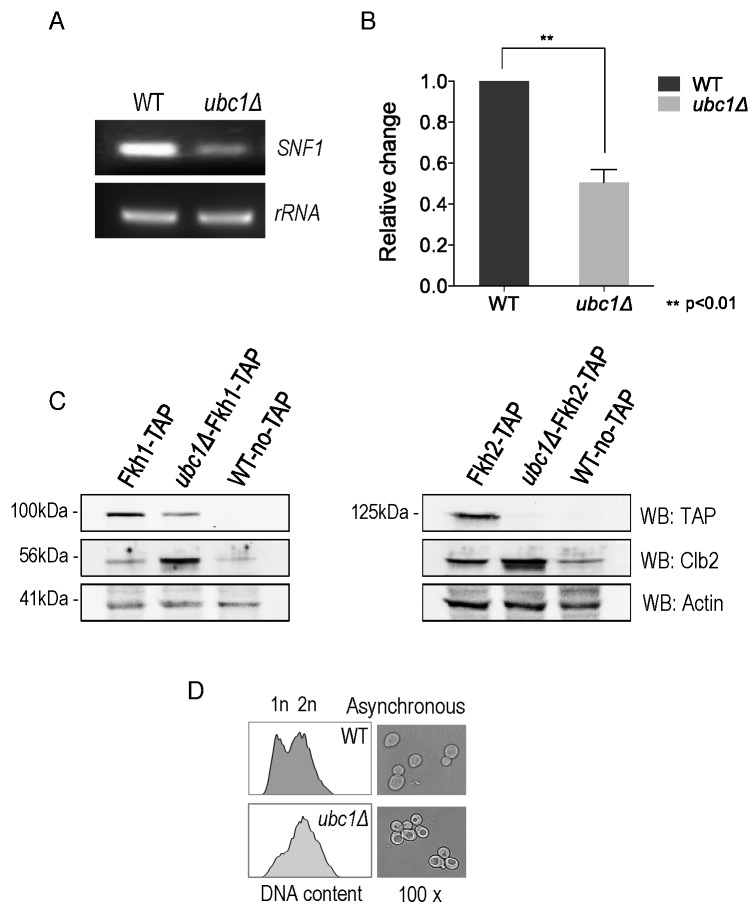
FIGURE 4: *SNF1* expression and Fkh protein abundance are decreased upon Ubc1 deletion. **(A)** *SNF1* and *rRNA* products from RT-PCR (26 cycles) were run on an agarose gel. RNA was isolated from isogenic WT and *ubc1*Δ strains that were grown to early log phase. **(B)** Quantitation of three biological repeats of (A) is shown with mean, SEM, and t-tailed significance (Prism 6.0). **(C)** The transcription factors Fkh1 and Fkh2 were endogenously TAP-tagged within the WT and *ubc1*Δ strains, and steady-state protein levels in 2% glucose (Fkh1-TAP left panel, 15 μg/lane, and Fkh2-TAP right panel 80 μg/lane) assessed by Western analysis. Clb2 is detected endogenously. **(D)** Light microscopy (100x objective) of an early logarithmic asynchronous culture showed the proportion of large-budded cells, with corresponding flow cytometry identifying the relative population of cells with replicated DNA (2n) in *ubc1*Δ compared to WT strains.

We addressed the possibility that *SNF1* transcription was decreased in the *ubc1*Δ strain, as an explanation for the decreased abundance of the Snf1 protein. RT-PCR of the *SNF1* gene revealed a clear decrease in transcription in the *ubc1*Δ strain (Fig. 4A), being approximately 50% that of the isogenic WT (Fig. 4B). We had previously found the yeast Fox orthologs, Fkh 1 and Fkh2, to be involved in *SNF1* gene expression 11 and asked if the absence of Ubc1 function was affecting Fkh1/2 activity, upstream of Snf1. In asynchronous cells, we observed that disruption of *UBC1* noticeably diminished Fkh1 protein abundance, while Fkh2 protein was essentially absent in the same strain even when significantly more protein (lysate) was tested (80 μg/lane for Fkh2 versus 15 μg/lane for Fkh1) (Fig. 4C). Fkh1 and Fkh2 are known to be transcriptionally regulated in synchrony with the cell cycle under the influence of a third forkhead member, Hcm1 19. Yeast strains deleted for *UBC1* have been found to be associated with cell cycle defects and to accumulate with large buds in late G2/M phase 16. Indeed, we found that the protein abundance of endogenous Clb2 was markedly elevated in the *ubc1*Δ strain as compared to WT, consistent with an accumulation of cells residing in G2/M phase when Clb2 levels are highest 20. To confirm this, flow cytometry of early logarithmic (OD_600_ of 0.4) asynchronous yeast cells from *ubc1*Δ strains demonstrated a significant inherent accumulation of cells with fully replicated DNA (2n) in G2/M (Fig. 4D). Light microscopy of cells representative of those undergoing flow cytometry show a heterogeneous population in WT with various bud sizes, yet a clear accumulation of large budded yeast cells in *ubc1*Δ, consistent with previous reports of G2/M arrest 16. The published report noted that *FKH1* and *FKH2* transcript levels are highest in G2/M phase and lowest in G1 19 was inconsistent with the decreased Forkhead protein levels we observed in the *ubc1*Δ strain partially stalled at G2/M, leading us to investigate this further.

### Snf1 protein, and glucose-responsive *SUC2* expression, levels are nearly reestablished by Fkh1 or Fkh2 overexpression in the *ubc1*Δ strain.

**Figure 5:**
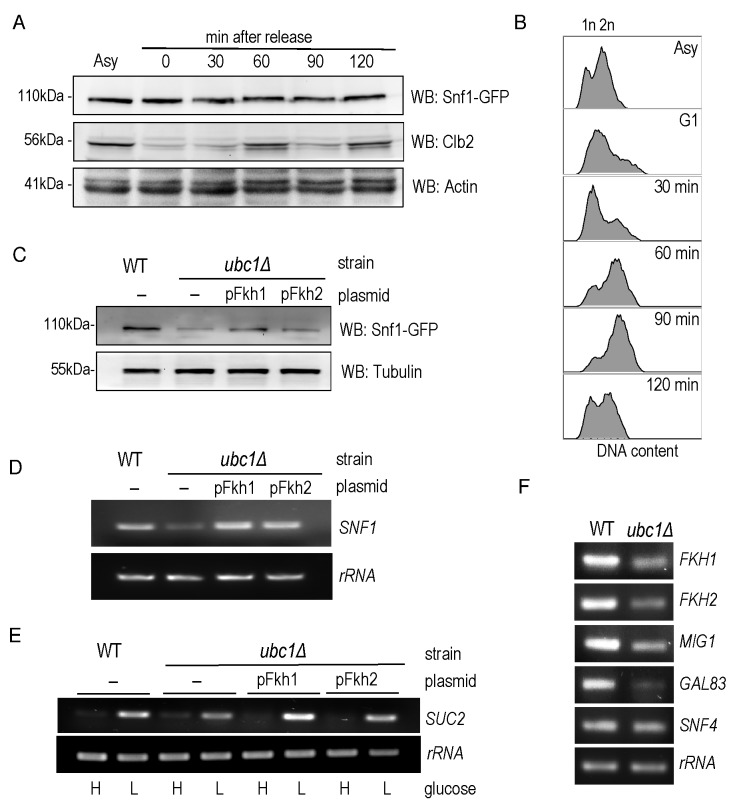
FIGURE 5: Snf1 protein and glucose-responsive *SUC2* expression levels are reestablished by Fkh1 or Fkh2 overexpression in the *ubc1*Δ strain. **(A)** α-factor arrest release of the WT yeast strain harboring an endogenous Snf1-GFP tag, with detection of Snf1-GFP and endogenous Clb2. Equal cell numbers were taken after release from G1 phase at the time points indicated, and Western analysis of the cell lysates as shown. **(B)** Corresponding flow cytometry of samples in (A) at the indicated time points, with unreplicated (1n) and replicated (2n) DNA abundance indicated. **(C)** Western analysis of steady-state Snf1-GFP protein levels in WT and *ubc1*Δ strains with or without 2μ plasmid expression of Fkh1 (pFkh1) and Fkh2 (pFkh2) in 2% glucose. **(D)** RT-PCR (26 cycles) of *SNF1* and *rRNA* expression in the *ubc1*Δ strain with and without independent 2μ plasmid expression of Fkh1 (pFkh1) and Fkh2 (pFkh2), with comparison to isogenic WT. **(E)** RT-PCR (26 cycles) of *SUC2 *expression under repressive (H: 2%) and activating (L: 0.05%) glucose levels with strains and plasmids as described in (D). **(F)** RT-PCR of genes encoding SNF1 kinase γ subunit (*SNF4*), β subunit (*GAL83*), *SUC2* repressor, *MIG1*, and the forkheads (*FKH1, FKH2*), comparing the *ubc1*Δ and WT strains. Asy: asynchronous. G1: α-factor arrest in G1.

Given that Fkh1 and Fkh2 can drive the expression of *SNF1* 11, and that Fkh1/2 gene expression fluctuates with the cell cycle, we asked if Snf1 protein levels fluctuate in synchrony with the cell cycle. There have been no reports of SNF1 kinase being regulated in a cell cycle dependent manner to the best of our knowledge. A G1 arrest-release experiment was performed in WT yeast to determine the inherent abundance of the Snf1 subunit protein level throughout the cell cycle. Fig. 5A reveals that Snf1 protein levels do not change as synchronized cells exit G1 and pass through the cell cycle, with fluctuations of Clb2 used as a surrogate marker for passage through the cell cycle 21. Further confirmation of successful synchronization comes from the flow cytometry analysis of these cells, showing the gradual shift of 1n to 2n (replicated) DNA (Fig. 5B). To ascertain if the decreased Snf1 abundance was a simple result of limited forkhead proteins, Fkh1 and Fkh2 were constitutively expressed in the *ubc1*Δ strain. The resulting Snf1 protein (Fig. 5C) and *SNF1* transcripts (Fig. 5D) are increased with Fkh1, which is not as apparent with Fkh2. Advancing one step further, we similarly investigated if Fkh1 or Fkh2 expression in the *ubc1*Δ strain restored the low-glucose-activated *SUC2* expression to that of WT levels. Fig. 5E shows that low glucose-induced expression of *SUC2* is at, or higher than, WT levels in the presence of the Fkh1 plasmid. To distinguish between etiologies underlying the decreased protein abundance of Fkh1 and Fkh2 (Fig. 4C), Snf4 and Gal83 (Fig. 3B) and Mig1 (Fig. 2D), we asked if the *ubc1*Δ deletion also affected their transcription. With the exception of Snf4, all RT-PCR products were of lower abundance in the *ubc1*Δ strain (Fig. 5F). Our data suggest that Fkh1/2 transcription of *SNF1* does not fully control Snf1 protein abundance in the *ubc1*Δ strain; despite compensated *SNF1* mRNA levels (Fig. 5D), Snf1 protein levels did not reach that of wildtype (Fig. 5C). Furthermore, constitutive plasmid expression of Snf1-HA within the *ubc1*Δ strain did not result in WT levels of Snf1 protein (Fig. S2A), suggesting that Snf1-HA is decreased in the *ubc1*Δ strain regardless of its endogenous transcription. Finally, we tested whether over-expression of the Snf4 γ-subunit would enhance Snf1 proteins levels, and concluded that it did not (Fig. S2B).

### Hcm1 is impaired in its cell cycle-dependent nuclear import, upon deletion of Ubc1.

**Figure 6:**
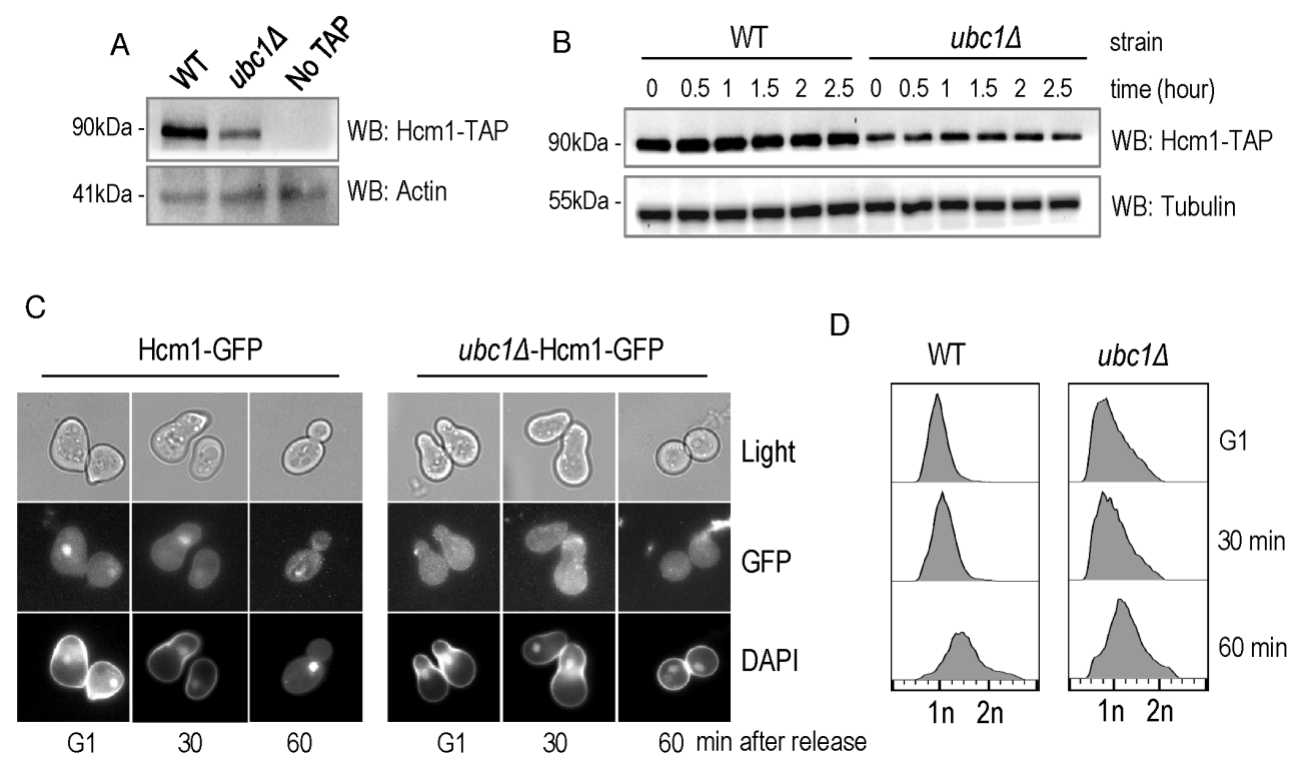
FIGURE 6. Hcm1 is impaired in its cell cycle dependent nuclear import upon deletion of Ubc1. **(A)** Equal numbers of early logarithmic phase cells from WT and *ubc1*Δ strains with or without an endogenous Hcm1-TAP tag were lysed followed by TAP Western analysis. **(B)** Assessment of Hcm1 protein stability over 3 hours in WT and *ubc1*Δ strains in the presence of cycloheximide, added at time (0), in 2% glucose. Equal cell numbers were removed at the indicated time points with 30 μg protein loaded. **(C)** Fluorescent microscopy of genomically integrated GFP-tagged Hcm1 expressed from its endogenous promoter in isogenic WT and *ubc1*Δ strains. Both strains were arrested in G1 followed by release, with cells collected at the indicated timepoints. **(D)** Flow cytometry analysis of cells collected at the timepoints in (A), highlighting the relative proportion of replicated DNA (2n) upon release from G1.

We sought a more detailed explanation for the decrease in Fkh1 and Fhk2 expression within the *ubc1*Δ strain, and focused on Hcm1, the forkhead family member known to regulate Fkh1 and Fkh2 expression in a cell cycle dependent manner 19. We tested both Hcm1 protein abundance, and its ability to shuttle between the cytosol and nucleus. We first found that the steady-state protein abundance of Hcm1 was modestly decreased upon disruption of the *UBC1* gene (Fig. 6A), although the protein appeared stable over an extended three-hour period (Fig. 6B). Hcm1 exhibits regulated nuclear import during G1 of the cell cycle, and we asked if the expected nuclear accumulation of Hcm1 in G1 22 is, in fact, disrupted by deletion of the *UBC1* gene. Fluorescent microscopy was used to determine endogenous Hcm1-GFP location within cells arrested in G1 and then released. We observed a clear nuclear accumulation in arrested WT cells, in direct contrast to a lack of nuclear-GFP signal in the *ubc1*Δ strain arrested in G1 (Fig. 6C). The morphology of the yeast cells, combined with the flow analysis (Fig. 6D) confirms that G1 arrest was successful in wildtype and *ubc1*Δ strains.

**Figure 7:**
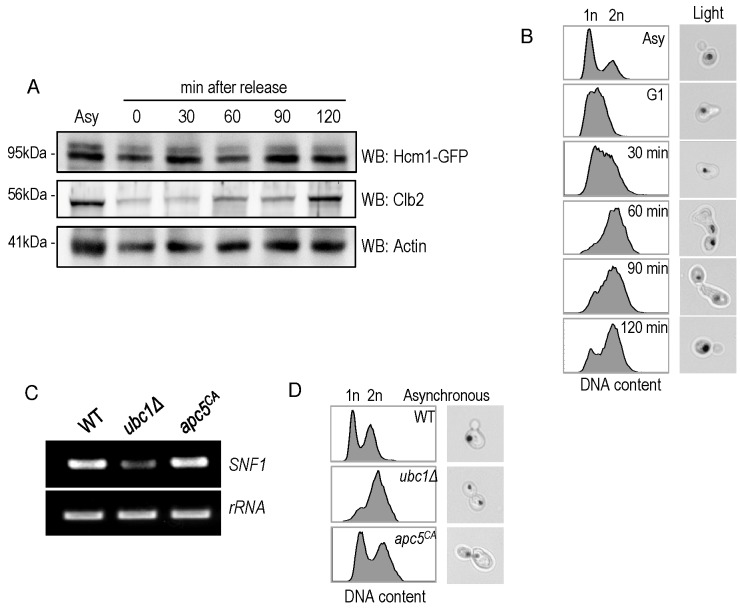
FIGURE 7: Hcm1 abundance is cell cycle dependent and cells are arrested at late M phase upon Ubc1 deletion. **(A)** α-factor arrest release of the WT yeast strain harboring an endogenous Hcm1-GFP tag, with detection of Hcm1-GFP and endogenous Clb2. Equal cell numbers were taken after release from G1 phase at the time points indicated, and Western analysis of the cell lysates as shown. **(B)** Corresponding FACS analysis of WT samples in (A) at the indicated time points, with unreplicated (1n) and replicated (2n) DNA content indicated. Light images (100x objective) indicate the cell morphology and nuclear position (propidium stained nucleic acid). **(C)** RT-PCR (26 cycles) of *SNF1* and *rRNA* expression in the *ubc1*Δ *apc5^CA^* strains, with comparison to isogenic WT. **(D)** Flow cytometry of early logarithmic asynchronous cultures demonstrating the relative population of cells with replicated DNA (2n) in *ubc1*Δ and *apc5^CA^* strains, compared to isogenic WT, with representative images of cell morphology and nuclear position. Asy: asynchronous.

To further analyze Hcm1 protein abundance differences between the WT and *ubc1*Δ strains, we compared the Hcm1 protein level throughout the cell cycle after G1 arrest and release in WT. Figure 7A demonstrated the cell-cycle fluctuations in Hcm1 protein levels, fully consistent with Hcm1 levels reported by others 23. 90 minutes after release, Hcm1 protein level reached maximum, followed by a decline at 120 minutes at which time Clb2 levels peaked, consistent with metaphase. Supporting this, flow cytometry analysis and microscopy images collected at the 120 minute timepoint are consistent with late mitosis/telophase; fully replicated (2n) and late mitosis (double budded, nuclei separated) (Fig. 7B). The doublet signal observed for the Hcm1-GFP Western analysis is not present in the Hcm1-TAP western blots, and is thus a non-specific artifact.

### Ubc1 impacts *SNF1* expression in an APC-independent manner.

Ubc1 is known to act with the APC to facilitate the metaphase-to-anaphase transition, and we were interested in determining whether Ubc1’s impact on transcription was APC-dependent, or -independent. We made use of yeast cells harboring the Apc5 subunit mutation (*apc5^CA^*), to test this. Figure 7C indicates that *SNF1* gene expression was not affected in the *apc5^CA^* temperature sensitive strain, and was decreased only in *ubc1*Δ cells. We next compared if the cell cycle arrest position and cell morphology between the *apc5^CA^* and *ubc1*Δ strains were similar. Early logarithmic, asynchronous, yeast cells from *ubc1*Δ and *apc5^CA^* strains were analyzed using flow cytometry and cells were imaged using propidium iodide staining of nucleic acid (Fig. 7D). Interestingly, the inherent arrest point of the *ubc1*Δ strain exhibited a distinctly different 1n/2n content, and nuclear positioning to that of *apc5^CA^* cells (Fig 7D). Together, these results clearly indicate that the role of Ubc1 on *SNF1* expression is independent of the APC.

### Schematic of Ubc1-dependent mechanisms and potential targets impacting SNF1 kinase activity.

**Figure 8:**
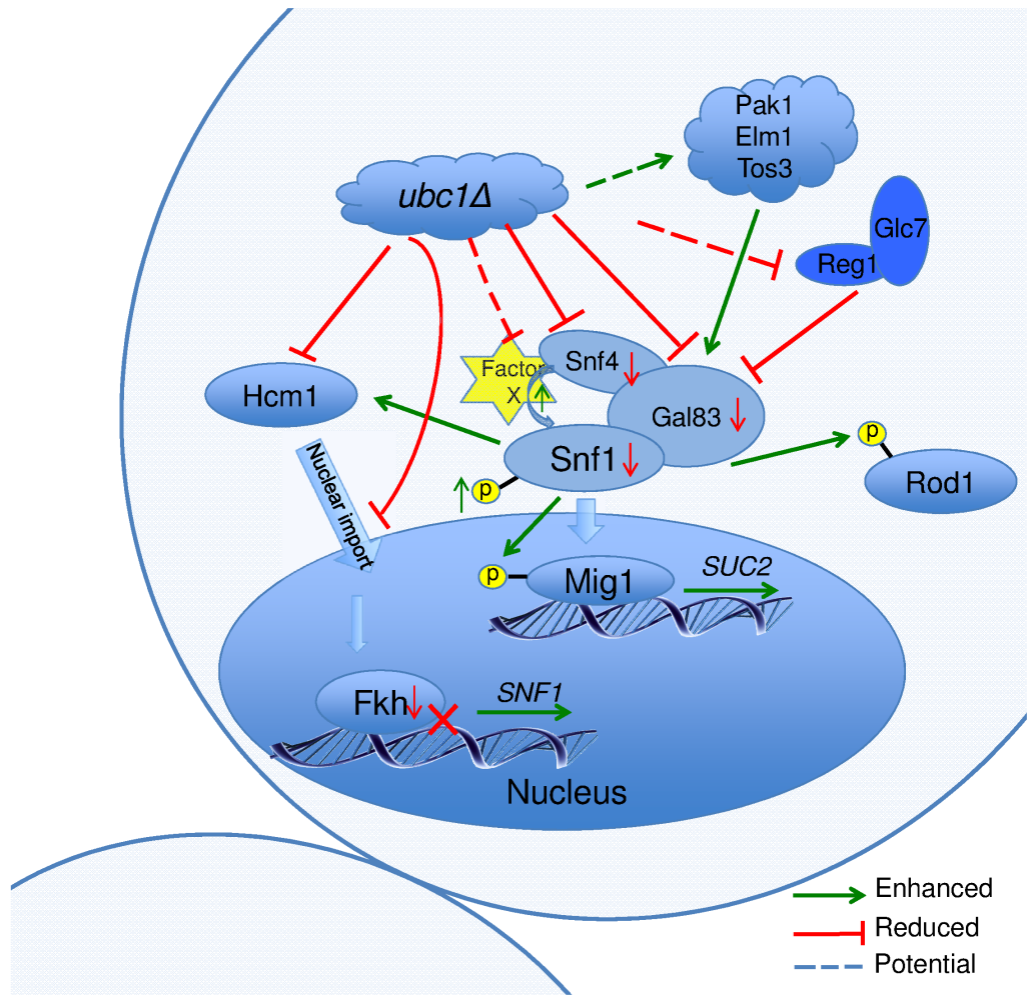
FIGURE 8: Schematic model of Ubc1-dependent mechanisms and their potential targets impacting SNF1 kinase activity. In the *ubc1*Δ strain, the Hcm1 protein fails to shuttle to the nucleus in a cell cycle dependent manner. The mechanisms is unknown, yet may include the failure of Ubc1 to degrade a cytosolic Hcm1 tether, or to provide an Ub-mediated import signal. The lack of nuclear Hcm1 results in impaired expression of *FKH1/2* genes, which required Hcm1 for expression. The decrease in Fkh1/2 protein in turn impedes the expression, and subsequent protein abundance, of Snf1. The Snf1 protein present, however, retained its functional ability to target cytosolic (Rod1) and nuclear (Mig1) proteins for phosphorylation, and itself be phosphorylated and translocated in response to activating conditions. There are enhanced allosteric associations between Snf1 and the regulatory Snf4 subunit in the absence of Ubc1 function, again by an unknown mechanism that may involve the removal of a moiety causing steric hindrance, Factor X. No obvious candidate protein is known that associates with the activated complex that would be stabilized by a loss of E2 activity.

Figure 8 graphically summarizes the observations made regarding the role of Ubc1 in SNF1 kinase function. In general, deletion of Ubc1 function resulted in decreased protein abundance of all three SNF1 kinase subunits tested, with transcriptional declines limited to Snf1 and Gal83 subunits, as Snf4 was not affected. The maintenance of WT-levels of Snf1 phosphorylation suggests a rebalancing of upstream kinase and phosphatase activities. The impaired *SNF1* expression is due to a simultaneous decrease in *FKH1* and *FKH2* expression and protein levels, ultimately appearing to arise from a failure of Hcm1 to accumulate in the nucleus to facilitate their transcription. The Ubc1 protein target involved in Hcm1 shuttling is not known. Enhanced allosterism within the SNF1 kinase complex may arise from the relative hyper-phosphorylation of Snf1, or from the loss of a Factor X that contributes to steric hindrance of subunit associations.

## DISCUSSION 

The SNF1 kinase/AMPK family of enzymes are tightly regulated, non-hormonal, sensors of stress and nutrient availability that facilitate adaptation of cellular pathways to maintain homeostasis. The yeast SNF1 kinase is a heterotrimeric Ser/Thr protein kinase complex that is activated, in part, by energy and nutrient limitations via an essential phosphorylation step. Understanding the intricacies of the subtle regulatory mechanisms controlling their activation and activity is also of great interest. Many fields, not the least of which is the potential benefit to human health, will advance with greater knowledge of regulatory targets to enhance the activity of these enzymes.

Through its kinase function, activated SNF1 kinase shifts the utilization of specific metabolic pathways in order to maintain cellular ATP levels 24. The SNF1 kinase is strongly evolutionarily conserved from yeast to humans, and fundamental mechanisms regulating SNF1 kinase activity in yeast have been proven to be likewise used in higher eukaryotes, including the essential phosphorylation on its α subunit, regulated dephosphorylation, allosteric subunit associations, and nuclear shuttling 12. In addition to these discrete steps in SNF1 kinase activation, it has also been reported that the catalytic α subunit of the yeast SNF1 kinase, Snf1 is polyubiquitinated and degraded 14, similar to an earlier report of this mechanism acting on the human ortholog, AMPK 25. Of note, we do not find evidence for inherent Snf1 protein instability and degradation in these experiments, irrespective of the kinase activation state or glucose availability (Fig 3A).

The covalent attachment of Ub to a target protein requires the concerted action of the Ub-activating (E1), Ub-conjugating (E2), and Ub-ligase (E3) activities 26, and specific protein targets are recognized and selected by specific E2/E3 combinations 16. The identity of the E2(s) required for the reported Ub conjugation to Snf1 has not been reported in yeast or in other eukaryotes to our knowledge, and as an approach to identify these factors, we systematically screened E2 deletion strains for effects on invertase activity, a SNF1 kinase-dependent event. Given the well-known consequence of protein stability changes upon ubiquitination, we had predicted that deletion of important E2 enzymes in Snf1-Ub targeting would create a stabilizing effect on Snf1, with the possibility of enhanced SNF1 activity due to Snf1 accumulation.

Here, we do not report the E2 involved in targeting Snf1 for ubiquitination. Instead, we report that deletion of the gene encoding the E2, *UBC1*, resulted in a ~50% reduction of SNF1 kinase activity that did not arise from deficiencies in glucose-regulated nuclear import or subunit associations, or in its ability to target cytosolic or nuclear targets for phosphorylation. Rather, the explanation resides in the noticeable decrease of Snf1 protein in the *ubc1*Δ strain, regardless of whether the growth conditions are activating or repressive for SNF1 kinase activity (Fig. 3A). It is interesting to note that, despite lower total Snf1 protein, a conspicuous and consistent enhancement of Snf1-Snf4 interactions throughout the 2-hybrid glucose gradient (Fig. 2C) and relative degree of Snf1 phosphorylation is maintained at near WT levels (Fig. 3A), yet was not capable of complementing for SNF1 kinase invertase activity. A simultaneous decrease of the β- (Gal83) and γ-(Snf4) subunits of the SNF1 kinase heterotrimer in this strain very likely contributes to this decrease in nuclear activity. This phenomenon of Snf1 hyperphosphorylation has been noticed previously 27 and has been proposed to be a compensatory mechanism to preserve SNF1 kinase function, with our results suggesting that it may also extend to allosterism. Decreases in the level of one subunit within SNF1 kinase have also been shown to correspond to decreases in the remaining subunits 28. Accordingly, we observed decreases in the γ subunit (Snf4) and the β subunit (Gal83) in the *ubc1*Δ strain (Figure 3B), perhaps as a means of preserving normal stoichiometry within the enzyme complex.

One of the major functions of ubiquitin conjugation is to target proteins for degradation by the proteasome 29. In contrast to protein degradation resulting from polyubiquitination through K48 chains, monoubiquitination (monoUb) of proteins has been shown to contribute to protein stability 30. Furthermore, ubiquitin conjugation can also have other important functions unrelated to protein degradation such as subcellular trafficking and protein associations 31. Ubc1 is known to contribute to K48 polyUb chain formation 16, whereas *in vivo* reports of intrinsic monoUb activity were not found. Interestingly, we found the Snf1 subunit to be stable for hours in both the WT and *ubc1*Δ strains (Fig. 3C), without detection of higher molecular weight bands under these physiological conditions to suggest Snf1 is polyubiquitinated. While the stability of Snf1 in the WT strain was initially unexpected, it is clear that the previously reported effects of Snf1 instability in response to SUMOylation and ubiquitination required the forced accumulation of polyUb chains attached to Snf1. This was enabled by genetic manipulation of the yeast strains, through disruption of the gene encoding the Ub-removal function, *UBP8*, and the resulting polyUb proteins that are targeted for proteasomal degradation 14,15. Otherwise Snf1 was not noticeably degraded.

Unexpectedly, the deletion of *UBC1* resulted in a substantial decrease in *SNF1* expression (Fig. 4A & B), and is likely a strong contributing cause for the low Snf1 protein levels in this strain. This correlates with a simultaneous decrease in Fkh1 and Fkh2 protein and transcript levels (Fig 4C and 5F). We had previously demonstrated that Fkh1, and to a lesser extent Fkh2, are necessary for *SNF1* expression 11. Constitutive expression of either Fkh1 or Fkh2 in the *ubc1*Δ strain returned *SNF1* transcript levels towards WT levels; Fkh1 was able to restore Snf1 protein levels and *SUC2* induction to a greater extent than Fkh2 alone (Fig. 5C & E).

The expression of *FKH1* and *FKH2* fluctuates with the cell cycle, and we asked if the inherent cell cycle defect in *ubc1*Δ might be affecting the low Fkh1/2 levels. The *ubc1*Δ strain is reported to arrest as large budded cells, which we also observed (Fig. 4D). Our flow cytometry analysis confirmed the high proportion of cells with replicated DNA indicating G2/M (Fig. 4D), and our propidium iodide staining for nuclei acid shows that the nuclei have clearly separated (Fig. 2A & B, Fig. 7E), altogether consistent with a late mitotic arrest. Lastly, the clear accumulation of Clb2 in asynchronous *ubc1*Δ cells is known to be a result of deficient E2 activity for the APC-dependent targeting of Clb2 for degradation, which is required for cell cycle progression through M into G1 16, explaining the arrest point of the *ubc1*Δ strain. The discrete differences between the *apc5^CA^* mutant and *ubc1*Δ cell cycle arrest points suggest that the Ubc1 cell cycle defect may be APC^Cdh1^ mediated. APC^Cdh1^ controls the M/G1 transition, whereas APC^Cdc20^ controls the metaphase/anaphase transition. General defects in APC function result in large budded cells with the nucleus aligned at the bud neck, as observed in Fig. 7D. Nonetheless, our observation indicates the cell cycle arrest in *ubc1*Δ cells is not a result of impaired APC activity. Furthermore, the expression of *FKH1/2* is normally high in G2/M in WT cells 19, yet we note an unexpected clear decrease of *FKH1/2* expression in the *ubc1*Δ strain that is enriched for G2/M phase cells. The answer appears to be due to Ubc1 affecting Hcm1, a third member of the Forkhead transcription factor family. Hcm1 is known to drive *FKH1/2* expression 19. A decline in Hcm1 protein abundance may explain the low Fkh1/2 protein levels in the absence of Ubc1, as Hcm1 was present at lower levels in the *ubc1*Δ strain, even compared to WT M-phase cells (peak expression of Hcm1) (Fig. 6A, Fig. 7A). What also became evident, however, was a specific defect in Hcm1 nuclear entry: Hcm1 is known to shuttle between the cytosol and the nucleus in a cell cycle manner, which controls its availability to the promoter regions of target genes, including *FKH1/2* 32. It is interesting to find that deletion of *UBC1 *severely impairs the nuclear accumulation of Hcm1 in G1 arrested cells (Fig. 6B). The role of ubiquitin in regulated nuclear import of target proteins has been well established 33,34. Accordingly, Ubc1 may normally target Hcm1 directly (or indirectly through an associated chaperone protein) for ubiquitination to promote import of Hcm1, or alternatively target a cytosolic tether for degradation. The identity of such a putative Ubc1 target controlling Hcm1 nuclear import was not revealed in this study, and we were unsuccessful in identifying Hcm1-Ub conjugates through co-immunoprecipitation experiments (data not shown).

It is also intriguing that a search of the literature revealed evidence that activated Snf1 is, in fact, a requirement for Hcm1 nuclear import 22. It is possible, therefore, that the decreased Snf1 protein abundance in *ubc1*Δ cells contributes to a negative feed-forward loop, impairing Hcm1 import and ultimately decreasing Fkh1/2 expression and protein abundance. This possibility deserves further investigation, as despite decreased Snf1 abundance, the relative activation state, as measured by its T^210^ phosphorylation, remains at wild-type levels.

We have discovered that the Ub-conjugating enzyme, Ubc1, is indirectly required for SNF1 kinase activity at the level of transcription. Ubc1 function was first associated with the cellular stress response 35, and later with vesicle biogenesis 36, endoplasmic reticulum associated degradation (ERAD) 37, and APC-dependent mechanisms for regulated cell cycle progression 16. What we describe here appears to be a novel function for Ubc1 that is independent of the APC ubiquitin ligase complex.

The schematic cartoon in Fig. 8 highlights our observations and several questions that surround the regulation of SNF1 kinase by Ubc1 activity. Despite WT levels of phosphorylation, nuclear import, and kinase activity on cytosolic and nuclear targets, there remains a deficiency in this activated complex to provide full *SUC2* expression. A simple explanation is that this may relate to the relatively low abundance of each of Snf1-Snf4-Gal83 protein subunits found in this heterotrimeric enzyme complex in the Ubc1 deletion strain. This decrease in Snf1 protein levels appears to be indirectly affected by Ubc1 through transcription via the Fkhs and Hcm1 (below) and not through Ub-mediated protein degradation. However, there appears to remain an overriding regulation of Snf1 protein levels that ultimately serves to maintain a stoichiometric balance between the three SNF1 kinase subunits. Secondly, the mechanism underlying the Ubc1-dependent failure of Hmc1 nuclear import is not known. Potential Ubc1 protein targets involved in Hcm1 shuttling are not reported, but may include a cytosolic tether that normally requires Ubc1 for degradation and subsequent release of Hcm1. Alternatively, Ub can serve as a nuclear import signal and this would be a novel role for Ubc1. Next, the enhanced allosterism noted within the SNF1 kinase complex in the *ubc1*Δ strain may arise from the relative hyper-phosphorylation of Snf1 at Thr^210^, or from the loss of a Factor X that contributes to steric hindrance of subunit associations. Disruption of the UBA domain in Snf1 also resulted in enhances allosteric associations 11, opening the possibility that this domain or protein face may have protein binding partners that physically regulate *SNF1* kinase activity through binding.

## Materials and Methods

### Creation of *UBC1* deletion strains

All strains utilized in this manuscript are listed within Table 1. The *ubc1*∆::*KanMX6* cassette was amplified using primers 500 bp up and downstream of *UBC1* and genomic DNA isolated from the *UBC1*/*ubc1*∆::*KanMX6* diploid strain (yTER301) as template. The entire cassette was individually integrated into the Fkh1-TAP, Fkh2-TAP, Mig1-TAP, Rod1-TAP, Snf4-TAP, Gal83-TAP, Hcm1-GFP, Hcm1-TAP, and 2-hybrid reporter strains, with primary selection for successful integrants being KanRes and final confirmation by PCR. Snf1-GFP *ubc1*Δ was created by crossing (*SNF1*-GFP::*HIS3* x *ubc1*∆::*HIS3*), tetrad dissection, scoring for markers, phenotypes, and confirmation by primer-specific PCR amplification.

**Table 1:** *Saccharomyces cerevisiae* strains used in this study.

**Strain (previous name)**	**Genotype**	**Reference or source**
yTER32 (PJ69-4A)	*MATa trp1-901 leu2-3 ura3-52 his3-200 gal4∆ gal80∆ LYS2::GAL1-HIS3 GAL2-ADE2 met2::GAL7-lac*	E. Craig
yTER301	*UBC1/ubc1::KanMX6*	W. Xiao
yTER305	yTER32 + *ubc1::KanMX6*	This study
yTER206	*MATa ade2 his3 leu2 ura3 SNF1-GFP::HIS3*	Life Technologies
yTER279 (MHY501)	*MATα his3-Δ200 leu2-3 112 ura3-52 lys2-801 trp1-1*	35
yTER277 (MHY509)	*MATα his3-Δ200 leu2-3 112 ura3-52 lys2-801 trp1-1 ubc1Δ::HIS3*	35
yTER299	yTER206 × yTER277	This study
yTH3926	*MATa ade2 his3Δ200 leu2-3 lys2Δ201 ura3-52 FKH1-TAP::HIS3*	T. Harkness
yTH3929	*MATa ade2 his3Δ200 leu2-3 lys2Δ201 ura3-52 FKH2-TAP::HIS3*	T. Harkness
yTER303	yTH3926 + *ubc1Δ::kanMX6*	This study
yTER304	yTH3929 + *ubc1Δ::kanMX6*	This study
yTER246	*MATa his3∆0 leu2∆0 met15∆0 ura3∆0 MIG1-TAP::HIS3*	Open Biosystems
yTER297	*MATa his3∆0 leu2∆0 met15∆0 ura3∆0 ROD1-TAP::HIS3*	Open Biosystems
yTER306	yTER246+ *ubc1Δ::kanMX6*	This study
yTER307	yTER297 + *ubc1Δ::kanMX6*	This study
yTER311	*MATa ade2 his3 leu2 ura3 HCM1-GFP::HIS3*	Life Technologies
yTER312	yTER311+ *ubc1Δ::kanMX6*	This study
yTER117	*MATa his3∆0 leu2∆0 met15∆0 ura3∆0 GAL83-TAP::HIS3*	Open Biosystems
yTER313	yTER117+ *ubc1Δ::kanMX6*	This study
yTER1	*MATa his3∆0 leu2∆0 met15∆0 ura3∆0 SNF4-TAP::HIS3*	Open Biosystems
yTER314	yTER1+ *ubc1Δ::kanMX6*	This study
yTER315	*MATα ade2 his3Δ200 leu2-3,112 lys2Δ201 ura3-52 apc5^CA^-PA::His5+*	39
yTER316	*MATa ade2 his3Δ200 leu2-3,112 lys2Δ201 ura3-52 apc10::KanMX6*	39
yTER187	*MATa ade2 his3Δ200 leu2-3,112 lys2Δ201 ura3-52*	This study
yTER70	*MATa ade2 his3Δ200 leu2-3,112 lys2Δ201 ura3-52 snf1Δ::KanMX6*	11
yTH1482	*MATα ade2 his3Δ200 leu2-3,112 lys2Δ201 ura3-52 mig1Δ::LEU2*	M. Carlson
yTER344	*MATa his3∆1 leu2∆0 met15∆0 ura3∆0 HCM1-TAP::HIS3*	GE Dharmacon
yTER346	yTER344+ *ubc1Δ::KanMX6*	This study

### Total protein extract and western blot analysis

Whole cell protein extracts from logarithmically growing cultures (no growth density greater than OD_600_ of 0.8) were prepared by a standard bead beat protocol 11 in the presence of RIPA buffer, and protease and phosphatase inhibitors (Sigma). Anti phospho-AMPK (Cell Signaling, 2535L), GFP (Covance, MMS-118P-500), Actin (Sigma, A4700, Lot005134), Clb2 (Santa Cruz, y-180), TAP (Open Biosystems, CAB-1001), and Tubulin (Sigma, 051M4771) antibodies were purchased and the chemiluminescent signal was captured on a VersaDoc (BIO-RAD) molecular imager (Quantity One 4.6.9).

### Invertase assay

Yeast strains were grown to early log phase in media that was consistent between comparison strains, with all comparison strains consistently in either rich (YPD) or minimal media, according to our published methods 18. Activity was normalized (value of 1) to that of WT in 0.05% glucose for each biological repeat. Statistical analysis utilized PRISM Version 6.0b software and 2-way ANOVA.

### Fluorescence microscopy

Fluorescence microscopy was used to detect GFP signal. To determine the subcellular localization of Snf1-GFP and Mig1-GFP, logarithmically growing cultures (OD_600_ < 0.8) were divided between non-activating complete media (CM), with 2% glucose versus activating (CM 5% glycerol) conditions for 30 minutes. Live cells were moved to mounting medium containing DAPI (Sigma) for immediate DNA visualization. To determine the subcellular localization of Hcm1-GFP, cells were arrested in G1 using α factor addition for 2 hours. Images were taken every 30 minutes following release of G1 arrest. Cells were viewed with an Olympus BX51 fluorescence microscope 100x objective equipped with an Infinity 3-1 UM camera. Images were collected using Infinity Analyse software version 5.0. A minimum of 125 cells for each strain and condition were consecutively scored for co-localization of the GFP-tagged subunits and DAPI nuclear staining.

### 2-hybrid analysis

The yeast WT 2-hybrid reporter strain (PJ69-4α, a gift from S. Fields) and the modified 2-hybrid strain *ubc1*Δ (*ubc1*∆::*KanMX6* cassette integrated into PJ69-4 α) were doubly transformed with pairs of empty vectors (-ve control: pGAD-C2 and pGBD-C2), or the same backbones expressing unmodified Snf1 and Snf4 subunits (+ve). 1x10^5^ cells from logarithmically growing cultures of each transformation set was repeatedly spotted down the glucose gradient of the slant plates 11, grown at 30°C until colonies were visible, and scanned. Freshly prepared warm liquid X-Gal agarose overlay medium 38 was layered to completely cover cells, solidified and incubated at 30°C and images scanned again after color development.

### mRNA expression analysis

RNA was isolated (RNAeasy Kit, Qiagen) from logarithmically (OD_600_ of 0.4) growing WT Snf1-GFP or *ubc1*Δ Snf1-GFP yeast strains that were transformed with pFkh1-GFP or pFkh2-HA plasmids followed by reverse transcription (QuantiTect Reverse Transcription Kit, Qiagen). cDNA (500 ng) was used as template in amplification reactions and equal reaction volumes were retrieved. Abundance was normalized to signal for ribosomal RNA (rRNA) at 26 cycles. VersaDoc (BIO-RAD) quantitation was obtained from RedSafe nucleic acid stain signal (FroggaBio).

### Cell cycle arrest

To arrest yeast cells in G1, 4 μg/mL α-factor was added to asynchronous early log phase (OD_600_ of 0.4) grown in YPD (pH 3.5) prior to experimental start. G1 arrested cell cultures demonstrated >90% of the cells had the expected G1 cell morphology under light microscopy. Cells were released by washing away α-factor and resuspended in fresh YPD. Equal volume cell samples were collected each 30 minutes for protein and fluorescent activated cell sorting (FACS) analysis. . The same Hcm1-GFP strain was used in Fig. 6C and D and Fig. 7A and B.

### Flow cytometry

Yeast strains of interest were grown in YPD to early logarithmic phase (OD_600_ of 0.4), 8x10^5^ cells (1 mL) were pelleted and washed with 50 mM Tris buffer (pH 8.0), then resuspended in 1 ml 70% ethanol overnight at room temperature to fix. Cells were pelleted and resuspended in 500 μL of 50 mM Tris buffer and digested with 10 μL 10 mg/mL RNase A at 37°C for 2 hours. Propidium iodide (10 μg/mL) was added to cells for ≥1 hour at room temperature away from light. Flow cytometry was performed using the EPICS® XL and data was analyzed with Flowjo software (v10.0.7).

### Cycloheximide experiment

To stop protein synthesis, 10 μg/mL cycloheximide was added to live logarithmic phase cells (OD_600_ < 0.8) in YPD medium. Equal cell numbers were collected every 30 minutes for cell lysate preparation and subsequent western blot analysis.

## SUPPLEMENTAL MATERIAL

Click here for supplemental data file.

All supplemental data for this article are also available online at http://microbialcell.com/researcharticles/the-ubiquitin-conjugating-enzyme-ubc1-indirectly-regulates-snf1-kinase-activity-via-forkhead-dependent-transcription/.
