# Supplementary material for: The ubiquitin-conjugating enzyme, Ubc1, indirectly regulates SNF1 kinase activity via Forkhead-dependent transcription
Source: Microb Cell. 2016 Nov 4;3(11):540–53. doi: 10.15698/mic2016.11.538 (PMC5349210; doi:10.15698/mic2016.11.538)
Supplement: Supplementary file 1 [file mic-03-540-s01.pdf]

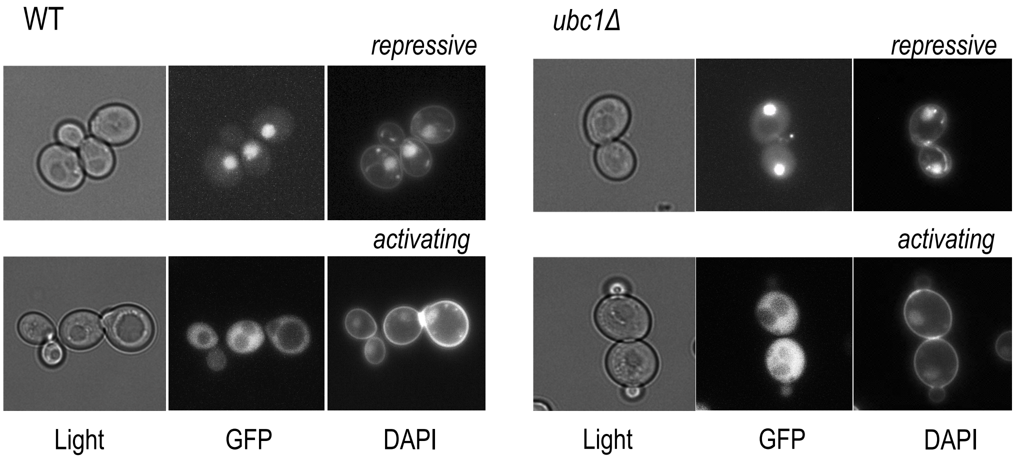

Figure S1. Mig1-GFP nuclear localization is not impaired in the *ubc1Δ* strain. Fluorescent microscopy of GFP-tagged Mig1, a *SUC2* transcriptional repressor constitutively expressed from a high copy 2μ plasmid in WT and *ubc1Δ* strains grown under repressive (2% glucose) or shifted to activating (5% glycerol for 30 minutes) conditions. Light: non fluorescent 100 x objective. GFP: green fluorescent protein epitope tag. DAPI: (4',6-Diamidino-2-Phenylindole, Dihydrochloride) a fluorescent DNA interchelator.

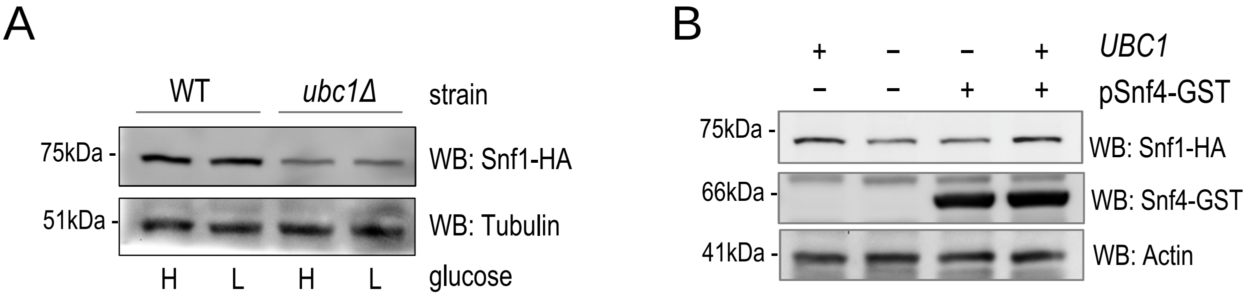

Figure S2. Snf1 protein level is decreased in the *ubc1Δ* strain, regardless of endogenous transcription. S2A, HA tagged Snf1 subunit was constitutively expressed from a high copy 2μ plasmid in WT and *ubc1Δ* strains. Transformed strains were divided, grown under activating (L) and repressive (H) glucose concentrations, and protein lysates taken for anti-HA Western analysis. **S2B**, plasmid expression of constitutively expressed Snf4-GST in the *ubc1Δ* strain (*UBC1* −) does not compensate for WT (*UBC1* +) protein levels of Snf1-HA (also plasmid expressed). Total protein was tested using anti-HA or anti-GST Western Blot. Anti-Tubulin and Actin western analysis was used as loading control. L: 0.05% low glucose; H: 2% high glucose.
